# Interactions of the Fungal Community in the Complex Patho-System of Esca, a Grapevine Trunk Disease

**DOI:** 10.3390/ijms232314726

**Published:** 2022-11-25

**Authors:** Laura Martín, Blanca García-García, María del Mar Alguacil

**Affiliations:** 1 Plant Protection Department, Scientific and Technological Research Centre of Extremadura (CICYTEX), 06187 Guadajira, Spain; 2 CSIC-Centro de Edafología y Biología Aplicada del Segura, Department of Soil and Water Conservation, Campus de Espinardo, 164, 30100 Murcia, Spain

**Keywords:** GTDs, grapevine, pathotrophs, Hymenochaetaceae, Botryosphaeriaceae, Illumina^®^ NGS, *Phaeomoniella chlamydospora*

## Abstract

Worldwide, Esca is a complex and devastating Grapevine Trunk Disease (GTD), characterized by inconstant foliar symptoms and internal wood degradation. A large range of fungal taxa have been reported as causal agents. We applied both culture-dependent and culture-independent methods (Illumina Technology and q-PCR) to investigate this concerning disease. Woods from vines with asymptomatic leaves and vines with leaf Esca symptoms were compared. Internally, different types of wood were found, from healthy wood with black necrosis to wood with white rot. A combination of leaf and wood Esca symptoms resulted in four experimental categories. Although there was no relation with symptoms, culture-independent mycobiome composition revealed *Phaeomoniella chlamydospora*, a GTD pathogen, as the most abundant species (detected in 85.4% of wood samples, with 14.8% relative abundance). Using TaqMan q-PCR, *P. chlamydospora* DNA was detected in 60.4% of samples (far from the 18.8% of positive results in the culture-dependent approach). There was a predominance of saprotrophs, even if their abundance was not affected by Esca symptoms. Concerning pathotrophs, the white rot development within grapevines was linked to the abundance of fungi belonging to the Hymenochaetaceae family. The Botryosphaeriaceae family was identified as an indicator for expression of Esca foliar symptoms. Lastly, the Aureobasidiaceae family was found to be a potential biocontrol agent for Esca, since it was most abundant in the control asymptomatic plants.

## 1. Introduction

*Vitis vinifera* is one of the most widely cultivated fruit crops with a great economic impact. A total global surface area of 7.5 million ha is under vines [1]. Grapevines are naturally colonized by a wide variety of microorganisms, including fungi, which interact with the plant, having either beneficial or pathogenic interactions. Severe economic losses are induced by fungal diseases. In this regard, Grapevine Trunk Diseases (GTDs) affecting perennial wood are currently considered the major threat to the sustainability of the grapevine sector [2]. In contrast with other fungal diseases in grapes (i.e., downy—powdery mildew), GTDs are plurennial, since phytopathogenic fungi inhabit the wood (but not the leaves) of the plant, causing gradual degradation and loss of functionality. The most standard approach, based on culture-dependent analysis of wood from GTD symptomatic vines, resulted in the isolation of a broad range of taxonomically unrelated fungi, which were assumed to be the causal agents of GTDs. In 2018, 133 fungal species, belonging to 34 genera, were reported to be associated with GTDs [3]. GTDs encompass a wide range of pathologies and syndromes, affecting young (black-foot disease, Petri disease, Botryosphaeria dieback and young grapevine decline) and mature vineyards (Eutypa dieback, Esca, Phomopsis dieback, Botryosphaeria dieback). All of these reduce plant longevity, ultimately causing plant death, and, thereby, reducing the economic profitability of the grape-wine industry [2,3,4,5]. Among the different GTDs, Esca is the most destructive worldwide. At the time of ripening, leaves of Esca-affected vines show an interveinal necrosis, known as “tiger stripes”, also called Grapevine Leaf Stripe Disease (GLSD), causing the drying of grape clusters, and, thus, affecting grape quality and composition [3,6,7]. Due to the simultaneous presence of causal agents and the inconstancy of leaf symptom expression, the diagnosis of Esca disease is complex, destructive and not practical. Moreover, reproduction of foliar symptoms by artificial inoculation has not always been successful [8]. To date, strategies to control Esca disease have rarely been effective [9]. The complexity of the etiology, epidemiology and diagnosis of Esca is a limitation to the sustainable and effective management of GTDs.

Culture-independent approaches have been applied to obtain a better understanding of the whole grapevine mycobiome. In recent years, different studies have worked on deciphering the microbiome at different levels, including aerial parts, roots–rhizosphere and vineyard soil. Microbial communities change along the vineyard’s vegetative cycle [10], as well as between cultivars and plant parts, and as a function of age [11,12]; these changes in the microbial community are also influenced by biogeographical factors and farming practices on vineyard soils [13]. Using both culture-dependent and culture-independent approaches, a large number of saprotrophs (905) and endophytes were reported in *Vitis vinifera*, highlighting the importance of applying both methods to study the fungal community composition [11,12]. However, the network of the complex mycobiome inhabiting the wood and related to foliar symptom manifestation of GTDs is still poorly understood. Few studies have compared the vine–wood mycobiome in terms of the expression of foliar Esca symptoms [14,15], and none of them investigated the trophic mode of the fungi. There is a significant lack of information related to the mode of life and function of fungal taxa that inhabit grapevines and their effect on the development of Esca symptoms.

Considering the above mentioned, we hypothesized that the interaction of different trophic modes resulting from the grapevine wood mycobiome is linked to Esca disease. Therefore, the aim of this work was to investigate the role of fungal communities in both wood degradation and foliar symptoms of Esca disease in grapevines. To this end, three specific objectives were developed:

(i) To elucidate differences in the fungal community inhabiting little degraded (healthy + black necrosis) or highly degraded (white rot) wood of grapevines showing esca foliar symptoms (symptomatic, Sy) or not (asymptomatic, As).
(ii) To determine the most informative point for detection of fungal infection within a vine. To this end, a comparison of the fungal population at four different parts (above the graft, A, strain cross, B, and right arm, C and left arm, D) of the vine was completed.
(iii) To compare both culture-dependent and culture-independent methods to identify and quantify the fungal species which colonized the grapevine wood. We characterized the wood mycobiome composition by ITS1 sequencing on an Illumina MiSeq instrument. Moreover, quantification of the fungus *Phaeomoniella chlamydospora* was achieved by using TaqMan qPCR. Molecular results were compared with the cultivable community of fungi to establish the most efficient methodology.

## 2. Results

### 2.1. Culture-Dependent Microbiological Analysis

**Figure 1:**
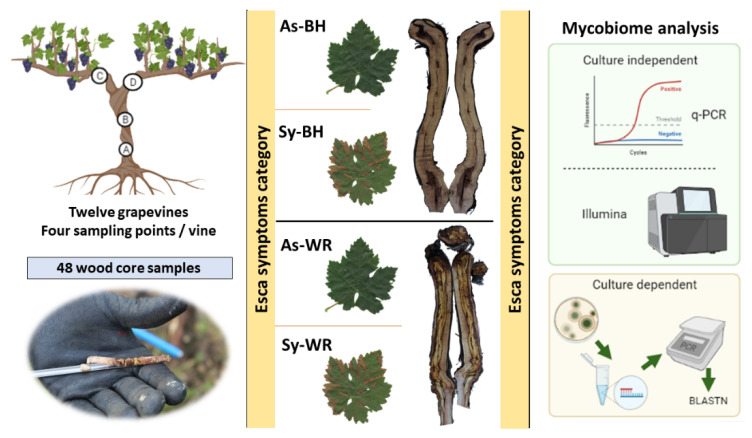
Graphic schedule of the material and methods used in the present work.

A total of 48 wood cores were segmented into six wood chips and cultured in a rich medium. Four categories related to foliar symptom manifestation (As vs. Sy) and type of wood symptom observed in the trunk (presence of white rot, WR, vs. presence of black necrosis with healthy wood, BH) were compared (Figure 1). 

**Figure 2:**
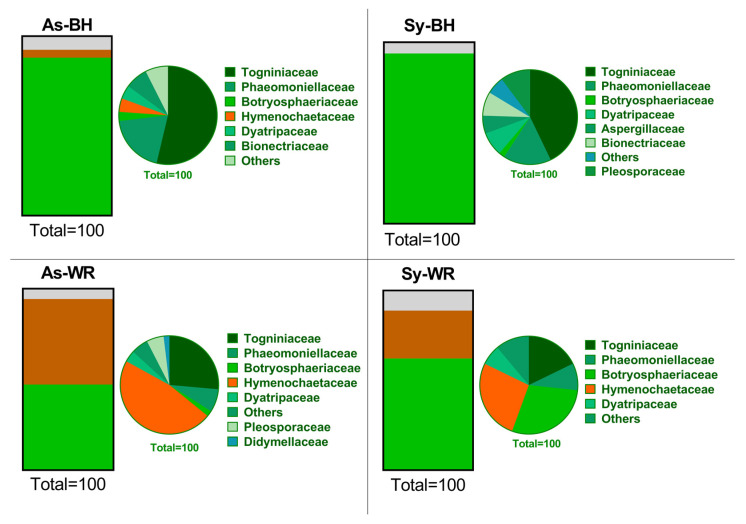
Relative abundance of fungal phyla and families resulting from culture-dependent microbiological analysis. Green color represents phylum Ascomycota, orange color represents phylum Basidiomycota, and gray color represents “unknown”.

There was no recovery of fungi from two samples belonging to Sy-BH and As-WR categories each. From the rest of the samples, we obtained 214 pure fungi cultures. The number of isolates was distributed as follows: 67 from the As-BH category, 53 from the As-WR category, 49 from the Sy-BH category and 45 from the Sy-WR category. At least one isolate linked to a morphotype, per wood sample, was selected to perform molecular analyses, resulting in a total of 81 fungal species identified at the genera or species level. Through both morphological characterization and further BLASTN analyses, GTD-related fungi were identified: Togniniaceae (all isolates identified as *Phaeoacremonium minimum*), Phaeomoniellaceae (all isolates identified as *Phaeomoniella chlamydospora*), Botryosphaeriaceae (with two species: *Dothiorella viticola* and *Diplodia seriata*), Hymenochaetaceae (with two species: *Fomitiporia mediterranea* and *Phellinus mori*), Dyatripaceae (with *Cryptovalsa ampelina* as the only species identified), Aspergillaceae (*Penicillium* spp.), Bionectriaceae (*Acremonium* sp.), Pleosporaceae (*Alternaria* spp.), Didymellaceae (*Epicoccum nigrum*) and “Unknown” when an accurate identification was not possible (i.e., low similarity of the ITS sequences < 97% in the BLAST analysis, or undetermined fungus). The relative abundance of Ascomycota in wood was reduced by the presence of Basidyomicota in categories As-WR and Sy-WR (Figure 2).

There was no effect of experimental factors (symptoms category/sampling point) on α-diversity estimator richness (S) and Shannon index (H’) (Supplementary Tables S1 and S2).

PERMANOVA analysis of the wood fungal community did not reveal a significant effect of the factor “sampling point” (*p* = 0.619), while the experimental factor symptoms category significantly impacted the fungal community structure (*p* = 0.001). The subsequent pairwise analysis showed that fungal communities belonging to the symptoms of the As-BH category were significantly different from those of the As-WR (*p* = 0.042) and Sy-WR (*p* = 0.018) categories.

Indicator families were found for three out of the four categories as follows: Togniniaceae for As-BH (*p* = 0.0149), Hymenochaetaceae for As-WR (*p* = 0.0026) and Botryosphaeriaceae for Sy-WR (*p* = 0.0061). No indicator species was found for any sampling point within a vine.

### 2.2. Culture-Independent Fungal Community Analysis

#### 2.2.1. Illumina^®^ NGS (Next Generation Sequencing) Results

Using Illumina technology, all 48 DNA samples were successfully sequenced and libraries were constructed. The total number of fungal sequences was 5,369,319. On average, 111,861 sequences, per wood sample, were obtained from the ITS region. High-quality sequencing of the fungal ITS region was clustered into 4174 fungal ASVs.

**Figure 3:**
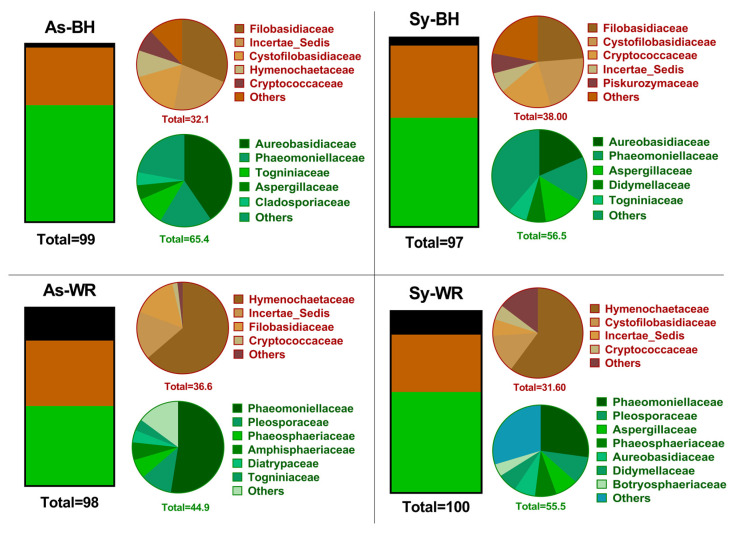
Relative abundance of fungal phyla and families resulting from culture-independent fungal community analysis. Green color represents phylum Ascomycota, orange color represents phylum Basidiomycota, and black color represents phylum Mortierellomycota.

The wood fungal community composition was mostly matched by phylum Ascomycota (58.09%) followed by phylum Basidiomycota (25.69%) and, to a lesser extent, by phylum Mortierellomycota (14.84%). The remaining phylum showed relative abundance below 1%. Figure 3 shows the mean relative abundance of sequences belonging to each phylum. The highest relative abundance of Ascomycota sequences was found in the As-BH category (65.43%). The relative abundance of Mortierellomycota sequences was increased by the presence of white rot in wood samples (18.52% and 12.71% in As-WR and Sy-WR categories, respectively) (Figure 3). A total of 203 families were identified across all wood samples, with Phaeomoniellaceae being the most abundant family (14.79%). The Esca pathogen *Phaeomoniella chlamydospora* was the only species within the Phaeomoniellaceae family, resulting in it being the most abundant species.

Esca symptoms had a significant impact (*p* < 0.05) on the α-diversity of wood mycobiota. The ANOVA showed that the fungal community from the Sy-BH category was significantly more diverse than the fungal community from the As-WR category (Table 1). The sampling point did not have a significant effect on α-diversity (Supplementary Table S3).

Regarding β-diversity, PERMANOVA analysis of the wood fungal community revealed a significant impact of the symptoms factor (*p* = 0.001) on fungal community structure, whereas the sampling point did not result in significant changes (*p* = 0.501). The subsequent pairwise analysis showed that fungal communities of the As-WR category were significantly different from those of As-BH and Sy-BH (*p* = 0.006) categories.

At the family level, indicator species were found for sampling points B (Microdochiaceae *p* = 0.0028; Lentitheciaceae *p* = 0.0480) and C (Cryptococcaceae *p* = 0.0356). When symptoms were compared, the ISA analysis showed 63 indicator families. The Aureobasidiaceae family was linked to the As-BH category (*p* = 0.0024). Three families were identified as indicators for the As-WR category: Hymenochaetaceae (*p* = 0.014), Rhynchogastremataceae (*p* = 0.046) and Xylariaceae (*p* = 0.0246). A total of 58 families were revealed as indicators for category Sy-BH (Supplementary Table S4) and Botryosphaeriaceae was detected as indicator for the Sy-WR symptoms category (*p* = 0.0315).

**Figure 4:**
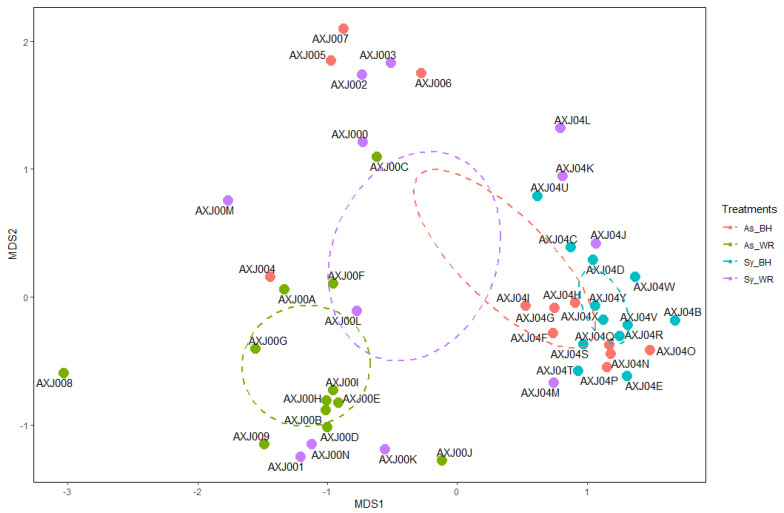
Ordination of wood fungal communities at the ASV level using a non-metric multidimensional scaling (NMDS) analysis on a Bray–Curtis dissimilarity matrix. Ellipsoids represent 95% confidence regions for each group (Site) mean.

The spatial ordination of the wood fungal community, resulting from the NMDS, returned a stress value of 0.19 and an R squared value of 0.963. The symptoms category influence on the fungal community of wood was distinguishable on the NMDS plot, in which the communities clustered in accordance with the differences between experimental categories revealed in the PERMANOVA post-hoc pairwise comparisons (Figure 4).

**Figure 5:**
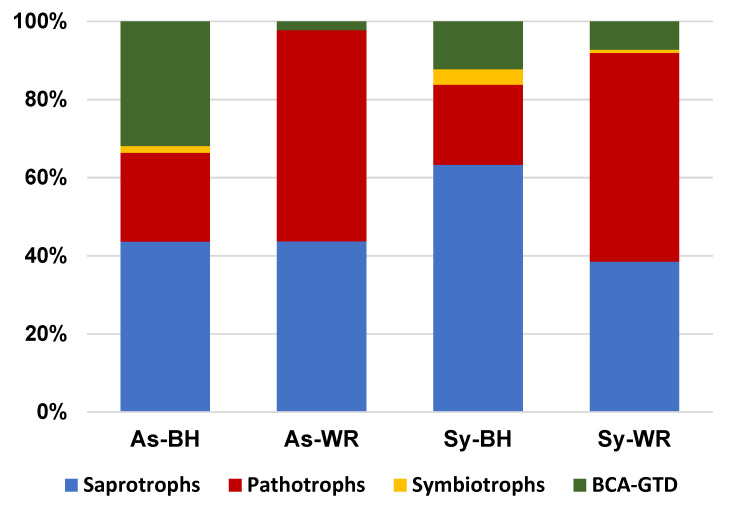
Relative abundance of trophic modes in wood samples. As-BH (foliar Esca asymptomatic vines with healthy or black necrosis in wood); As-WR (foliar Esca asymptomatic vines with white rot in wood); Sy-BH (vines with Esca foliar symptoms and healthy or black necrosis in wood); and Sy-WR (vines with Esca foliar symptoms and white rot in wood).

After careful revision according to both FUNGuild and Põlme databases [16,17], as well as other grapevine fungal literature, we were able to assign each of the 622 prevalence ASVs to their trophic mode and guild status (Supplementary Table S5). The analysis resulted in the following classification: 300 saprotrophs; 226 pathotrophs and 66 symbiotrophs. Moreover, there were 30 ASV candidates to act as GTD biocontrol agents (BCA-GTDs). In general, saprotrophs were predominant in the fungal community of grapevine wood, whereas symbiotrophs were scarce (Figure 5).

The scarcity of symbiotrophs could be a direct result of their lifestyle, as these fungi may be more active in degrading dead plant matter. To better understand how trophic modes and guilds are likely to respond to inputs of organic matter, we analyzed both the total percentages of nitrogen and carbon in wood samples. Although a significant effect of the symptoms category on wood composition was not found, a tendency linking white rot to higher C and N content was observed (Table 2).

Most saprotrophs are reported as endophytes in grapevines [11,12,18]. ANOVA analysis of the relative abundance of the ASVs classified as saprotrophs showed no changes among symptoms (*p* = 0.1019). As opposed to what was observed with saprotrophs, the symptoms category had a significant effect on the relative abundance of ASVs with pathotroph behavior (*p* = 0.0006). Symbiotrophs were represented by 10 ASVs assigned to the ericoid mycorrhizal guild belonging to genera *Leohumicola* (2 ASVs) and *Oidiodendron* (8 ASVs), plus 56 ASVs of the ectomycorrhizal guild (Supplementary Table S5). ANOVA analysis showed a major proportion of symbiotrophs in the Sy-BH category (*p* = 0.0056). The BCA-GTDs group was dominated by *Aureobasidium*, as well as mycoparasites *Trichoderma*, *Fusicolla*, *Keithomyces*, *Metapochonia*, *Papiliotrema* and *Tremella* (Supplementary Table S5). Moreover, a significantly higher abundance of BCA-GTDs was found in the As-BH category (*p* = 0.0006).

**Figure 6:**
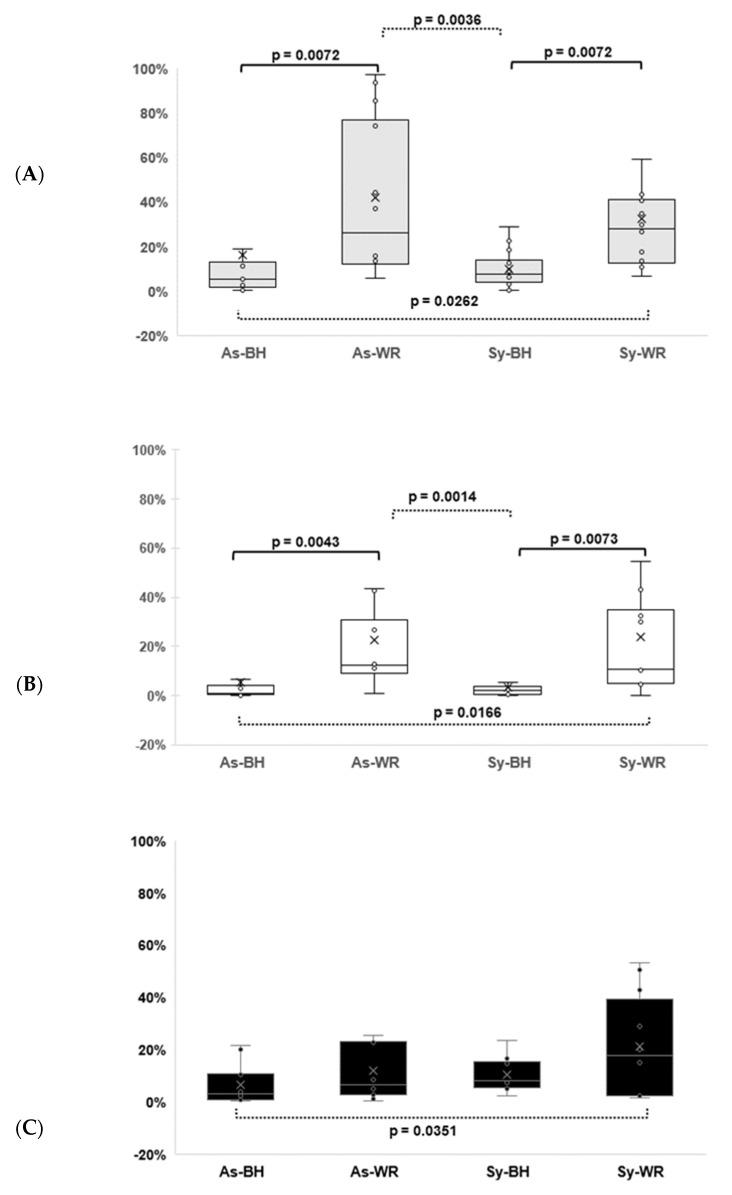
Relative abundance of three different guilds of pathotrophs (calculated for the total 622 fungal ASVs that were assigned to a trophic mode) in grapevine wood of the four studied symptom categories. (**A**): GTD-related pathogens; (**B**): fungi causing white rot; and (**C**): plant pathogens. Means and standard errors (*n* = 12) are shown for symptoms (As-BH, As-WR, Sy-BH and Sy-WR). Significant differences between two categories are shown with *p*-values < 0.05, according to the Mann–Whitney (Wilcoxon) test.

Regarding pathotrophs, a total of 226 ASVs were found in this work. Two functional guilds were assumed as having an impact on the Esca patho-system: GTD-related fungi (128 ASVs) and those causing white rot (86 ASVs). Most of the ASVs causing white rot belonged to the Hymenochaetaceae family, except genera *Ganoderma, Gymnopus, Peniophora, Uncobasidium* and *Xenasmatella* (Supplementary Table S5). All ASVs identified as members of the Hymenochaetaceae family (77 ASVs) were considered to belong, at the same time, to both guilds GTD-pathotrophs and white rot (Supplementary Table S5). The remaining pathotrophs (89 ASVs), plus the genera causing white rot, belonged to genera different from Hymenochaetaceae and 9 ASVs were classified as belonging to the plant-pathogen guild, having a total of 98 ASVs (Supplementary Table S5/Figure 6C). All pathotrophs together were significantly higher in theAs-WR (54.07 ± 0.086) and Sy-WR (53.45 ± 0.069) categories than in the As-BH (22.73 ± 0.083) and Sy-BH (20.49 ± 0.025) categories (*p* = 0.0006). Figure 6A (based on abundance of GTD pathotrophs) and Figure 6B (based on abundance of white rot guild) show the same pattern. Figure 6C shows that the relative abundance of plant-pathogens was significantly lower in the As-BH symptoms (which could be considered as control), than in the most Esca affected category (Sy-WR).

#### 2.2.2. Results of *P. chlamydospora* Quantification by q-PCR

The obtained averaged values for standard regression curve parameters resulting from five independent technical replicates were: y = −3.61 (±0.25)x + 39.44 (±2.26); with a R2 = 0.988 (±0.008). Standard errors are written in parentheses. According to the MIQUE guideline [19], the calculated PCR efficiency was 92.89% (±10.43) and only samples showing a frequency of positive amplification > 95% (six positive detections out of two independent experiments, DNA extraction, with three replicates each) were considered positive. Under the experimental conditions, the lowest limit of *P. chlamydospora* quantification was determined to be 540 fg. When using total DNA extracted from wood as the template, a total of 29 out of 48 wood samples were positive (60.42%) for the presence of Pch DNA. Naturally infected wood provided, on average, 23,290.5 fg/reaction of *P. chlamydospora* DNA. There was no significant effect of the symptoms on the amount of Pch quantified by q-PCR (*p* = 0.126).

### 2.3. Biological Conclusions

The inner wood of vines hosts a great fungal diversity with a predominance of the saprotrophic mode of life. In this environment, the species *Phaeomoniella chlamydospora*, which is a pathogen related to GTDs, was found to be the most abundant one. White rot development within grapevines was linked to the abundance of fungi belonging to the Hymenochaetaceae family. Moreover, when both white rot and Botryosphaeriaceae species co-existed, foliar symptoms of Esca might be expressed. By contrast, a major abundance of the Aureobasidiaceae family seemed to have biocontrol activity and might prevent white rot in wood and Esca foliar symptoms in grapevines.

## 3. Discussion

### 3.1. Comparison of Both Culture-Dependent and Culture-Independent Methods

There is a high richness of fungal taxa in grapevine cultivation. Until a decade ago, characterization of mycobiomes was usually made by culture-dependent analysis. The advantages of the traditional techniques are that they allow isolation of live microorganisms and, moreover, when combined with PCR amplification of partial genes and subsequent BLASTN analysis, they also allow accurate identification of taxa. The GTDs complex is a factor of particular interest, in, for example, the case of the Botryosphaeriaceae family and the involvement of *Phaeoacremonium* (Pm) species in Esca disease, since partial sequences of the ITS region is not enough to identify isolates at the species level [20,21,22,23]. Using both culture-dependent and culture-independent methods, Jayawardena et al. (2018) provided a worldwide checklist of 905 fungal taxa for *Vitis* [12]. In the traditional approach, they were able to identify the taxa to species level, while in the culture-independent method they were frequently able to identify the taxa at the family or genus level. A similar limitation of the culture-independent approach was found in our study, as 53 of the most frequent ASVs were identified as Hymenochaetaceae at the family level, but not at the genus or species level (Supplementary Table S5). Jayawardena et al. (2018) reported that the fungal taxa overlap at the genus and species level between the traditional and culture-independent approaches was relatively low [12]. In the present work, the number of ASVs obtained with Illumina was 4174, while the culture-dependent obtained 11 ASVs, representing only 0.26%. There is no doubt that richness and diversity of the fungal community was higher using the independent culture approach (i.e., comparison of Table 1 with Supplementary material Table S1; comparison of Supplementary material Table S2 with Table S3). This study was in line with the statements of previous studies that recommend the use of traditional techniques to accurately identify taxa and propose culture-independent methods to obtain a better understanding of the organisms that are present in a host [12]. In our study, the pathogen *P. chlamydospora* was very useful to compare the power of three different tools applied to diagnose naturally infected grapevine wood. Positive detection in the 48 wood samples was 18.8% in the culture-dependent approach, and 60.4% and 85.4% in the culture-independent approach for q-PCR and Illumina^®^, respectively. However, it is worth highlighting that the most outstanding results of our research were consistent between the two techniques. There was a similar pattern in Figure 2 (culture-dependent) and Figure 3 (culture-independent), except for phylum Mortierellomycota. Moreover, the ISA was consistent in recognizing the Hymenochaetaceae family as an indicator for the As-WR symptoms and the Botryosphaeriaceae family as an indicator for the Sy-WR symptoms, using both culture-dependent and culture-independent approaches. Our research demonstrates that Illumina^®^ NGS is a valuable tool in deciphering the Esca patho-system, as it increased the sensitivity (number of Pch positive samples) and accrual of information on the mycobiome network.

### 3.2. Pathogenetic Role of P. chlamydospora and Botryosphaeriaceae, Fungi Associated to GTDs

GTDs are widely recognized as a major threat to the wine sector, as they severely compromise the longevity and productivity of grapevines by increasing production costs [2]. The worldwide annual financial cost of the replacement of dead plants due to GTDs was quantified at over 1.132 billion euros [4]. Esca is a major grapevine trunk disease that heavily affects vineyards in the Northern hemisphere [8,14,24]. In the literature, the principal taxa associated with Esca disease are *P. chlamydospora* and *Phaeoacremonium* species, two tracheomycotic and ascomycetes, as well as basidiomycete *Fomitiporia mediterranea*, especially common in Europe [3,5,7,24]. To a lesser extent, Botryosphaeriaceae and Dyatripaceae fungi, mostly associated with other GTDs (i.e., Botryosphaeria dieback and Eutypa dieback), have also been reported in Esca symptomatic vines [8,14,15].

It is known that fungal GTD phytopathogenic species are isolated from necrotic and degraded wood tissue, which is the unquestionable niche for them. Few works have also reported the presence of these causal agents in healthy wood and foliar asymptomatic vines, especially in young vines [4]. One of the most puzzling points about these fungi is that, although they can cause necrosis when they are inoculated in vines, the foliar symptoms are frequently absent [14]. In general, pathogenicity assays have demonstrated their role as causal agents of wood symptoms, whereas they often fail in leaf symptoms reproduction [8]. Different hypotheses may help in explaining this puzzling point, such as the liberation of phytotoxic compounds by fungi and the role of abiotic stress in the Esca patho-system [7]. However, Bortolami and co-authors recently demonstrated that Esca does not result from decreases in water potential [25]. According to the above mentioned, the involvement of these fungi in GTDs is being questioned as a unique cause of Esca disease. Altogether, the research demonstrated that there is a gap between fungal infection in wood and the manifestation of Esca symptoms in leaves. The exact link between foliar and wood necrosis remains unclear. Only Maher et al. (2012) studied different types of internal necrosis in grapevine wood to determine the relationships between necrosis and the severity of foliar Esca symptoms. Results of a logistic model indicated that white rot in the cordons was the best predictor for the chronic form of Esca [26]. Understanding interactions of the fungal community inhabiting the wood with leaf symptoms expression is a major concern in the Esca patho-system. Two previous works have investigated the grapevine wood mycobiome of vines that expressed or did not express Esca foliar symptoms: one using metabarcoding (SSCP Single Standard Conformation Polymorphism) [14] and one using Illumina Seq. technology [15]. Two innovative aspects distinguish our investigation from previous research: (i) this is the first study to report differences among experimental categories that considered both foliar and wood Esca symptoms; (ii) our findings not only described taxon abundance but also delved into the trophic network.

Bruez and colleagues (2014) reported that, regardless of the sampling time, the mycobiomes of asymptomatic and symptomatic plants were not significantly different. In their study, trunk wood tissues were apparently healthy (non-necrotic), and white rot was present only in cordons. Species of Hypocreaceae and Botryosphaeriaceae were the most abundant among the 48 genera they isolated [14]. It is worth pointing out that results from the present work found the Botryosphaeriaceae family to be an indicator for Esca leaf symptom manifestation (the Sy-WR category) using both methods (culture-dependent and culture-independent).

In Portugal, the characterization of the wood mycobiome in a vineyard affected by Esca, resulted in a richness of 289 taxa [15]. The mycobiome composition of wood in leaf-symptomatic canes and that of plants showing no Esca leaf symptomatology was similar. By contrast, the spatial analysis revealed differences in diversity, evenness and taxa abundance between canes and other perennial parts of the vine [15]. In the present study, the sampling point showed no effect on the mycobiome community. This fact could be due to the fact that we did not analyze wood from only one year (canes). In accordance with our results, the Esca-associated species *Phaeomoniella chlamydospora* dominated the Ascomycota fungal community at 25.8% and 14.8% of relative abundance in del Frari et al. (2019) and in the present work, respectively. However, in both studies, no relationships between expression of foliar symptoms and abundance of *P. chlamydospora* fungus was found, suggesting new insights on their role as causal agents of Esca. In the past, species-specific PCR primers were designed for TaqMan^®^ q-PCR, which easily detected Pch [19]. This study evaluated the application of this q-PCR tool to diagnose Esca using a minimal invasive sampling of wood. According to the MIQUE guideline [27], good technical results were achieved. Moreover, *P. chlamydospora* DNA was detected in 60.4% of total samples (far from the 18.8% of positive results in the culture-dependent approach). Our results support the potential use of q-PCR for the detection of Pch pathogen in plant material [19]. Even though *P. chlamydospora* was the most abundant species, the utility of q-PCR as a non-cultured method for Esca diagnosis remains uncertain, since Pch was not detected as an indicator species for the expression of Esca leaf symptoms.

### 3.3. Basidiomycota and Hymenochataceae Family as Key of Pathotrophs

Regarding the other relevant results, the Basidiomycota fungal community was dominated by Hymenochaetaceae, which were considered pathotrophs, since they were lignicolous, causing white rot. This is the first work to report the trophic mode of the 622 most representative ASVs found in wood in a vineyard affected by Esca. The relative abundance of pathotrophs was significantly higher in the symptoms of the As-WR and Sy-WR categories than in the As-BH and Sy-BH categories. This fact could be explained by two guilds of pathotrophs (namely GTD-related fungi and those causing white rot) (Figure 6A,B) and because 59.4% of GTD-pathotrophs belonged to the Hymenochaetaceae family, thus also causing white rot. Results further confirmed the patterns observed within PERMANOVA (showing significant differences between As-WR and both As-BH and Sy-BH symptoms). Similarly, the ISA analysis identified the Hymenochaetaceae family as indicator for the As-WR category, using both culture-dependent and culture-independent approaches. In conclusion, differences were not linked to the expression of foliar Esca symptoms (low abundance of pathotrophs in Sy-BH category), although our results confirmed a pathogenic role of this family causing white rot in the Esca patho-system. A review on white rot fungi (Hymenochaetales) and Esca in grapevines was recently published, suggesting that the simultaneous presence of Hymenochaetales and tracheomycotic fungi is a pre-requisite for leaf symptoms [8]. Our results agree with this hypothesis and also reveal the Botryosphaeriaceae family in the manifestation of leaf Esca symptoms.

*F. mediterranea*, which is a member of the Hymenochaetaceae family, has been reported to be involved in Esca proper, and to be abundant in Europe [3,5,7,8,24]. Since sodium arsenite was banned (in the year 2003 in Europe), there are no effective methods to control Esca. In a recent work [28], the impact of sodium arsenite on wood microbiota of Esca-diseased grapevines proved that the relative abundance of *F. mediterranea* significantly decreased after treatment (−90%). These results strongly suggest that the effect of sodium arsenite (the only known efficient molecule able to control Esca foliar symptoms) is due to the elimination of *F. mediterranea* from white rot necrotic tissues, allowing saprobic fungi to colonize it [28]. Similar results were found when trunk surgery was performed, reducing foliar Esca symptoms [29]. In the present work, *F. mediterranea* was not found to be an indicator of foliar Esca symptoms, perhaps due, in part, to the limitation of the Illumina NGS technology to identify Hymenochaetaceae taxa at the species level.

### 3.4. New Insights on Biocontrol and Microbiome Interaction

The major challenge in Esca disease is to find a sustainable control strategy. Pinto and colleagues (2018) demonstrated the potential role of *Aureobasidium pullulans* to prevent the infection caused by *Diplodia seriata* (a Botryosphaeriaceae spp. involved in GTDs, mainly Botryosphaeria dieback) both in vitro and in grapevine models [30]. Our results indicate that the Aureobasidiaceae family is linked to the As-BH category (i.e., the asymptomatic category in leaves and healthy wood that can be considered control). This is in line with the importance of this family as a control agent for Esca, or at least as a promoter of grapevine resilience under the study conditions of this work. Potential beneficial plant interactions between *Bacillus*, *Streptomyces* bacteria and GTDs have been reported [2]. In a recent study, a stronger correlation with foliar symptoms was shown by bacteria compared to the mycobiome [31]. A synergistic interaction between the fungus *F. mediterranea* and *Paenibacillus* bacteria was recently discovered, suggesting that interactions might enhance grapevine wood degradation [32]. Future research is needed to better understand the role of bacteria to promote or avoid Esca under field conditions, as well as its interaction with the mycobiome in grapevine wood.

## 4. Materials and Methods

### 4.1. Vineyard

The vineyard plot was located in “Tierra de Barros”, which is known to have clay soils, in Badajoz (Extremadura region), southwestern Spain (38.616911, −6.555444). Experimentation was conducted on the cultivar Tempranillo. It was planted in 2002 (16 years old) grafted onto 110-Richter rootstock, trained according to a double Royat cordon system and oriented north–south with a density of 1667 vines per hectare. In 2018, climate conditions registered an average annual temperature of 16.4 °C (average maximum temperatures = 22.1 °C; average minimum temperatures = 10.5 °C) and the cumulative annual precipitation was 575.8 mm. The Köppen–Geiger climate classification was Csa, i.e., warm temperatures with a dry and hot summer [33]. There was very low irrigation support. Expression of foliar Esca symptoms of 500 grapevines was visually monitored in 2018 (5 July) at ripening time. We registered the number and position of: (i) vines showing no Esca symptoms, namely asymptomatic (As), and (ii) vines developing Esca symptoms on leaves which were considered diseased plants and symptomatic (Sy). Among Sy plants, two types of symptoms were differentiated: grapevines with apoplexy and vines showing the typical “tiger stripe” leaves. Dead plants and faults (i.e., dead plants before 2018 already uprooted at that moment) were also counted. Severity of Esca disease was calculated according to McKinney’s index [34] by using a numerical scale as follows: 0 = no Esca symptoms in leaves; 1 = plants showing typical “tiger stripe” leaves; 2 = grapevines with apoplexy; 3 = completely dead plants (without green growth). McKinney’s index expressed the percentage of Esca severity by using the following formula: MI = [Σ(R × N)] × 100/H × T, where R = disease rating, N = number of plants with this rating, H = the highest rating, and T = total number of plants counted (500). The Esca severity according to McKinney’s index was 6.6. The incidence of Esca symptoms ranged from 6.4% of vines displaying GLSD leaves, 1.6% of vines with apoplexy symptoms and 3.4% of grapevines dead in 2018 or before. The total loss of plants due to Esca disease was 11.4% of plants.

Soil samples (*n* = 3) were taken at three randomly symptomatic vine-sample points to a depth of 0.15 m. A fraction of soil was air-dried, ground and sieved to 2 mm for chemical analysis. Characteristics of the top soil were as follows: clay texture with 52.33 ± 0.51% of clay; 18.18 ± 0.05% of silt and 29.49 ± 0.45% of sand; pHwater (1: 2.5) 7.9 ± 0.24; electrical conductivity (1:5) = 75.63 ± 0.63 uS/cm; and organic matter = 0.64 ± 0.02%.

### 4.2. Wood Sampling

On 5 February 2019, 48 wood samples were collected right before the vineyard plot was totally uprooted to plant a new one, due to low productivity. Twelve plants (six Sy grapevines and six As grapevines) were randomly selected (on the basis of annotations made on 5th July, 2018) to perform a microbiome study. To determine the most informative point for fungal microbiome research within a vine, four wood cores were taken (Table 3).

Wood samples were collected in a non-destructive way using a Pressler’s increment borer designed for use in hard wood (two grooves; inner diameter = 5.15 mm, length = 300 mm) (Haglöf, Sweden). Samples were taken from the following: (A) above the graft-union (located approximately 11 ± 1 cm above the soil); (B) at the ‘strain cross’, i.e., the cross part right before the two arms separation (located approximately 40 ± 1 cm above the soil); and at the (C) right arm and (D) left arm (Figure 1). On each sampling point, the bark was removed and the gimlet was disinfected with ethanol (70% *v*/*v*), before and after extracting each core of wood (5 mm in diameter and approximately 60 mm long). Then, the samples were immediately placed in sterile 50 mL falcon tubes until further microbiological and molecular analysis. Finally, all twelve plants were longitudinally cut to assess the presence of white rot, black necrosis or healthy wood in the trunk. The twelve plants were categorized in four experimental categories according to both development of Esca foliar symptoms observed at ripening time (July) and wood symptoms observed in the trunk when uprooted after the longitudinal cut. Four categories were namedas follows: (1) foliar asymptomatic grapevines with healthy and/or black necrosis along the trunk (As-BH); (2) foliar asymptomatic grapevines with white-rot along the trunk (As-WR); (3) grapevines expressing Esca foliar symptoms with healthy or black necrosis along the trunk (Sy-BH); and (4) grapevines expressing Esca foliar symptoms and white rot along the trunk (Sy-WR). There were three biological replicates (grapevines) per category of experimental (Table 3) (Figure 1).

To better understand how the fungal community and trophic modes were linked to inputs of the wood composition, determination of the total content of carbon and nitrogen were analyzed according to the standard UNE-EN ISO 16948:2015 for solid biofuels. The chemical analysis was carried out in triplicate using 100 mg of a mixture of wood power from four independent wood samples per category of symptoms. The results were presented as mean ± standard errors of three repetitions per category.

### 4.3. Culture-Dependent Microbiological Analysis

Fungi described as potential causal agents of Esca were isolated from wood. Sample cores were surface sterilized by submersion in sodium hypochlorite and three consecutive washes in sterile water for 3 min each under aseptic conditions. Twelve small wood chips per core were obtained. All wood types (healthy, black necrosis and white rot) were proportionally represented and cultured in a rich medium (malt extract agar and potato dextrose agar, both amended with 0.25 mg/mL chloramphenicol). Plates were incubated in the dark at 25 °C for 7 days. The observed fungi were transferred individually to fresh medium, and pure cultures were obtained to conduct morphological and molecular identification. Fungal isolates were first identified by morphological characterization (based on mycelia color, growth ratio and conidia characteristics, when possible) in the following groups, according to the literature [20,21,22,23,24,35,36,37]: Hymenochaetaceae family, Botryosphaeriaceae family, Dyatripaceae family, genera *Phaeoacremonium spp*. and *Phaeomoniella chlamydospora* species. Several representative isolates from each group were selected to obtain molecular identification. Total genomic DNA was isolated and amplified from fresh mycelium using the REDExtract-N-Amp Kit (XNAP) (Sigma, St. Louis, MO, USA), following the manufacturer’s instructions. Hymenochaetaceae and Dyatripaceae molecular identification was performed by sequencing the internal transcribed spacer (ITS) region ITS1-5.8S-ITS2, using primers ITS4 and ITS5 [38]. In this work, we used the methodology described by Alves et al. (2005) [39], as well as amplification of the Elongation Factor, using primers EF1-728F and EF1-986R [40] for identification of Botryosphaeriaceae fungal isolates. In the case of *Phaeoacremonium* species, species-specific primers Pal1N and Pal2 [41] were used to confirm the presence of *Phaeoacremonium minimum* species. In addition, molecular identification was conducted according to previous report analyses of the β-tubulin (BT) and calmoduline (CAL) genes [21]. Finally, fungal isolates of *Phaeomoniella chlamydospora* was confirmed both by species-specific PCR amplification using primers Pch1 and Pch2 [41] and sequencing analysis of the ITS region. All PCR amplifications were performed using a T100™ thermal cycler (BioRad Laboratories, Inc. Hercules, CA, USA). Primer sets were supplied by Metabion International AG (Planegg, Germany) and sequences were obtained using an Applied Biosystems 3500 Series Genetic Analyzer (Thermo Fisher, Waltham, MA, USA), with technical and human support provided by the Facility of Bioscience Applied Techniques of SAIUEx (financed by the University of Extremadura, Junta de Extremadura, MICINN, FEDER and FSE). The sequences were then read, edited and analyzed using the BLASTN tool (https://blast.ncbi.nlm.nih.gov/ (accessed 1 April 2022)) according to previous work [21], in order to identify homologous sequences.

### 4.4. Culture-Independent Fungal Community Analysis (qPCR and Illumina Technology)

#### 4.4.1. DNA Extraction

A total of 0.025 g of powdery wood was used for the DNA extraction using the Danagene Microbiome Soil DNA kit (Danagene), according to the manufacturer’s instructions and adapted by Biome Makers. The yield and purity of the DNA was measured by Qubit and then stored at −20 °C until use.

#### 4.4.2. Amplification, Library, Illumina Sequencing and Bioinformatics Processing

To characterize fungal microbial communities associated with wood samples, the internal transcribed spacer (ITS) marker regions were selected. The libraries for ITS were prepared using a two-step PCR, as described by [42,43]. Libraries were obtained by amplifying the ITS1 region using BeCrop^®^ custom primers, (patent WO2017096385). All libraries were prepared following the two-step PCR Illumina protocol, and these were subsequently sequenced on an Illumina MiSeq instrument (Illumina, San Diego, CA, USA) using 2 × 250 paired-end reads.

Primers were removed from paired-end reads using Cutadapt [44]. Then the trimmed reads were merged with a minimum overlapping of 100 nucleotides. Next, the sequences were quality filtered by Expected Error with a maximum value of 1.0 [45]. After quality pre-processing, reads with single nucleotide differences were iteratively clustered together to form ASVs (Amplicon Sequencing Variants) using Swarm [46]. De novo chimeras and remaining singletons were subsequently removed [47]. Finally, taxonomy was assigned from ASVs using a global alignment with 97% identity, against a curated reference database from UNITE 8.3 for ITS sequences [48,49].

#### 4.4.3. Quantification of the Esca Causal Agent *Phaeomoniella chlamydospora* by qPCR

Fungal DNA quantification of the Esca pathogen *Phaeomoniella chlamydospora* was assayed by q-PCR. Reactions were performed using the TaqMan system previously described [19]. To generate a standard curve for the quantification of Pch, seven 1:10-fold serial dilutions obtained from pure genomic DNA were prepared, according to Martín et al. (2012) [19]. The gDNA from isolate CBS 229.99 was used for standard curves. In all PCR experiments, a non-template control (NTC; water) was included to test PCR performance. Assays were performed as two independent experiments, with three replicates for both standards and samples. To establish the limit of detection (LOD), which was defined as the minimum level at which the analysis could be reliably detected with a probability of 95%, the lower gDNA concentration (108 fg of DNA/reaction per Pch) had nine replicates. From the wood samples, two independent DNA extractions were analyzed in two independent experiments.

Reactions were conducted in a StepOne PlusTM Real-Time PCR System (Applied Biosystems) using the following standard program: 20 s at 95 °C plus 40 cycles of 1 s at 95 °C and 20 s at 60 °C. The final reaction volume was 20 µL containing 1× Premix Ex Taq Probe qPCR (Takara), 0.3 µM each primer, 0.15 µM TaqMan probe, and 2 µL of DNA template. The obtained mean values of the threshold cycles (Ct) were plotted against the logarithm of the DNA concentration in the reaction to construct the standard curve and to calculate the DNA quantity of Pch in the wood samples. In addition, the slope of each standard curve and PCR efficiency was estimated. Interpretation of qPCR results were performed following the MIQE guidelines, as described by Bustin et al. [27].

### 4.5. Statistical Analysis

The results obtained using both methodologies (culture-dependent and culture-independent analyses) were compared and discussed. Alpha diversity was calculated through richness, Shannon’s index (H’) and Pielou evenness index for each sample (*specnumber* and *diversity* functions, VEGAN package in R). The influence of the sampling point and symptoms on the diversity indices was determined by one-way ANOVA analysis. Subsequent comparisons among means were performed using the Tukey HSD (Honestly Significant Difference) test, calculated at *p* < 0.05.

To assess β-diversity, analyses of the resulting fungal communities were performed in the R programming environment (v3.6.1; R Core Team, 2019; Methods S3). To avoid bias by differences in sequencing effort, we normalized the data by rarefying the samples to 18,256 sequences. A non-metric multidimensional scaling (NMDS) analysis, which resulted in the spatial ordination of samples according to the composition and structure of the fungal community, was performed. To better understand whether the fungal communities inhabiting grapevines were significantly affected by the experimental factors named sample point and symptoms, a permutational multivariate analysis of variance (PERMANOVA) was conducted with the Adonis function in vegan [50]. The PERMANOVA was calculated with the Hellinger-based distances as a measure of dissimilarity and 999 permutations. A post-hoc pairwise comparison with Bonferroni correction was conducted on the PERMANOVA results.

Indicator species analyses (ISAs) were conducted on the virtual taxa relative abundances with the index species package for R [51]. We considered sampling point and symptoms as classifier factors. This analysis identified taxa associated eith one of the categories of a given factor by calculating an Indicator Value (IndVal) [52] and its significance.

After the number of reads was rarefied, a prevalence filter was applied, thus, only ASVs detected in at least 10% of samples were retained, obtaining 622 ASVs, with only associations with a *p*-value ≤ 0.05 being preserved. A careful revision was performed to classify ASVs into trophic mode and functional guilds using both FUNGUILD v.1. and Põlme et al. (2021) databases [16,17]. Relative abundances of trophic groups were calculated and plotted per symptoms category. Relative abundances were compared by a simple analysis of variance. The LSD (Least Significance Difference) test was used for discriminating among means. Comparison of two symptoms categories was made with the non-parametric Mann Whitney–Wilcoxon test. Differences at *p* < 0.05 were considered significant. Statistical procedures were carried out using StatGraphics Centurion XVI software (Manugistics Inc., Pockville, MD, USA).

## 5. Conclusions

Understanding interactions of the fungal community inhabiting the wood with expression of leaf symptoms is a major concern to develop control strategies in the Esca patho-system. The pathogen *P. chlamydospora* was the most abundant species in the wood of grapevines, although there was no relation with symptoms. Among the three methodologies, culture-independent methods (q-PCR and Illumnia seq.) were more sensitive to detect Pch, since the smallest number of Pch positive samples was found using culture-dependent analysis. The potential BCA Aureobasidiaceae family was more abundant and it was identified as indicator of the control asymptomatic vines (As-BH). The relative abundance of two types of pathotrophs (namely GTD-related fungi and those causing white rot) were significantly higher in vines with white rot symptoms in wood (Sy-WR and As-WR categories). The Botryosphaeriaceae family was identified as indicator for expression of Esca foliar symptoms (Sy-WR category), while the Hymenochaetaceae family was detected as indicator of asymptomatic leaves (As-WR).

## Figures and Tables

**Table 1:** Alpha-diversity estimation of the fungal community resulting from the culture-independent analysis of 48 wood samples.

Symptoms	S	H’	J’
As-BH	213.25 ± 32.99 b	2.33 ± 0.35 ab	0.45 ± 0.06 ab
As-WR	138.00 ± 12.88 b	1.82 ± 0.12 b	0.37 ± 0.02 b
Sy-BH	405.09 ± 57.92 a	3.41 ± 0.32 a	0.57 ± 0.05 a
Sy-WR	202.50 ± 39.12 b	2.67 ± 0.30 ab	0.52 ± 0.06 ab
ANOVA (*p*-values)	8.64 (0.0001)	5.40 (0.0030)	3.71 (0.0182)

S: Richness; H’: Shannon’s index; and J’: Pielou evenness index. Mean ± standard error, *n* = 12. Significance of symptoms category effect is shown by F-values and *p*-values. Mean values within a column followed by different letters are significantly different according to Tukey test (*p* < 0.05).

**Table 2:** Percentage of carbon (C) and nitrogen (N) resulting from the grapevine wood samples.

Symptoms	C (%)	N (%)
As-BH	27.20 ± 1.99	0.17 ± 0.06
As-WR	36.03 ± 5.46	0.23 ± 0.03
Sy-BH	27.33 ± 1.30	0.19 ± 0.02
Sy-WR	28.17 ± 2.69	0.20 ± 0.03
ANOVA (*p*-values)	1.70 (0.244)	0.42 (0.747)

Mean ± standard error, *n* = 3. Significance of symptoms category effect is shown by F-values and *p*-values.

**Table 3:** List of the four categories of symptoms related to foliar symptoms manifestation (As vs. Sy) and type of wood symptoms observed in the trunk (presence of white rot, WR, vs. presence of black necrosis with healthy wood, BH) compared in this work. List of grapevines (*n* = 12) and list of wood core sampling points (48). GC: Grapevine code; SP: Sampling point.

Symptoms Category	Foliar Esca Symptoms	Wood Symptoms Observed in Trunk	GC	SP
As-BH	Asymptomatic	Healthy wood and black necrosis	C04	A, B, C, D
			C26	A, B, C, D
			D86	A, B, C, D
As-WR	Asymptomatic	White rot	D43	A, B, C, D
			E08	A, B, C, D
			E60	A, B, C, D
Sy-BH	Symptomatic	Healthy wood and black necrosis	D96	A, B, C, D
			D80	A, B, C, D
			C01	A, B, C, D
Sy-WR	Symptomatic	White rot	C40	A, B, C, D
			C16	A, B, C, D
			C41	A, B, C, D

## Data Availability

The sequence files were submitted to the NCBI Sequence Read Archive Repository (www.ncbi.nlm.nih.gov/sra (accessed 6 October 2022)) and are accessible in the Bio Project PRJNA887603.

## Supplementary Materials

The following supporting information can be downloaded at: https://www.mdpi.com/article/10.3390/ijms232314726/s1.
