# Supplementary material for: Interactions of the Fungal Community in the Complex Patho-System of Esca, a Grapevine Trunk Disease
Source: Int J Mol Sci. 2022 Nov 25;23(23):14726. doi: 10.3390/ijms232314726 (PMC9736784; doi:10.3390/ijms232314726)
Supplement: Supplementary file 1 [file ijms-23-14726-s001.zip › ijms-2045643-supplementary.pdf]

Table S1. Alfa-diversity estimation of the fungal community resulting from the cultured analysis of wood samples. Factor symptoms category.

Symptoms	S	H′	J′
As-BH	1,83±0,27	0,45±0,14	-
As-WR	1,55±0,21	0,33±0,12	-
Sy-BH	1,55±0,21	0,34±0,12	-
Sy-WR	1,50±0,20	0,31±0,12	-
ANOVA (p-values)	0,48 (0,6946)	0,24 (0,8667)	

S: Richness; H′: Shannon's index and J′: Pielou evenness index. Mean ± standard error, n=12 (n=11 for Sy-BH and As-WR). Significance of treatment effect is shown by F-values and p-values.

Table S2. Alfa-diversity estimation of the fungal community resulting from the cultured analysis of wood samples. Factor sample point.

Sample Point	S	H′	J′
A	1,75±0,25	0,44±0,14	-
B	1,42±0,15	0,26±0,10	-
C	1,70±0,26	0,41±0,15	-
D	1,58±0,23	0,33±0,12	-
ANOVA (p-values)	0,45 (0,7203)	0,42 (0,7383)	

S: Richness; H′: Shannon's index and J′: Pielou evenness index. Mean ± standard error, n=12 (n=10 for C). Significance of treatment effect is shown by F-values and p-values.

Table S3. Alfa-diversity estimation of the fungal community resulting from the non-cultured analysis of wood samples. Factor sample point.

Sample Point	S	H′	J′
A	277,42±73,69	2,75±0,43	0,50±0,06
B	255,92±39,84	2,89±0,29	0,53±0,04
C	227,58±38,68	2,28±0,21	0,43±0,04
D	197,92±30,28	2,30±0,33	0,44±0,05
ANOVA (p-values)	0,51 (0,6802)	0,92 (0,4391)	0,94 (0,4299)

S: Richness; H′: Shannon's index and J′: Pielou evenness index. Mean ± standard error, n=12. Significance of treatment effect is shown by F-values and p-values.

Table S4. Indicator species analysis (ISA) for Sy-BH category at family taxa level.

**Significance**	***		**		*
Agaricaceae	0.0004	Amanitaceae	0.0019	Bartaliniaceae	0.0245
Arachnomycetaceae	0.0006	Ascobolaceae	0.0049	Chaetosphaeriaceae	0.0140
Bionectriaceae	0.0006	Bankeraceae	0.0072	Coniochaetaceae	0.0370
Boletaceae	0.0003	Chaetomiaceae	0.0032	Dermateaceae	0.0342
Clavicipitaceae	0.0003	Clavulinaceae	0.0022	Didymosphaeriaceae	0.0163
Cortinariaceae	0.0005	Cordycipitaceae	0.0096	Hydnaceae	0.0453
Gloniaceae	0.0005	Discinaceae	0.0067	Hygrophoraceae	0.0331
Lasiosphaeriaceae	0.0001	Gymnoascaceae	0.0054	Hypocreaceae	0.0316
Lipomycetaceae	0.0005	Helotiaceae	0.0034	Melanogastraceae	0.0269
Myxotrichaceae	0.0003	Helvellaceae	0.0076	Melanommataceae	0.0201
Pyronemataceae	0.0010	Herpotrichiellaceae	0.0065	Mucoraceae	0.0261
Russulaceae	0.0004	Hyaloscyphaceae	0.0013	Pannariaceae	0.0474
Sporormiaceae	0.0008	Hymenogastraceae	0.0017	Piskurozymaceae	0.0156
Stachybotryaceae	0.0003	Inocybaceae	0.0051	Schizoporaceae	0.0480
Trichocomaceae	0.0007	Lycoperdaceae	0.0049	Thelephoraceae	0.0295
Tricholomataceae	0.0004	Nectriaceae	0.0031	Torulaceae	0.0346
Tubeufiaceae	0.0002	Omphalotaceae	0.0037	Trichosporonaceae	0.0359
Umbelopsidaceae	0.0002	Onygenaceae	0.0011		
		Pseudeurotiaceae	0.0018		
		Schizoparmaceae	0.0082		
		Sebacinaceae	0.0027		
		Trichomeriaceae	0.0020		
		Trimorphomycetaceae	0.0057		

Significance: *** p-value < 0.001; ** p-value < 0.01; * p-value < 0.05

Table S5. Trophic mode and guild classification of the 622 most representative ASVs: 297 saprotrophs (S); 229 pathotrophs (P): classified as plant-pathogen (PP), Grapevine Trunk Diseases associated fungi (GTDs) and those causing white rot (WR); 66 symbiotrophs (Sy) and 30 potential biocontrol for GTDs (BCA).

TM	Phylum	Order	Family	Genus	Nº ASVs	Nº spp.	PP	GTDs	WR	Ecto	Ericoid	References
S	Ascomycota	Capnodiales	Cladosporiaceae	*Cladosporium*	8	3 + sp.	-	-	-	-	-	[12,16]
S	Ascomycota	Chaetosphaeriales	Chaetosphaeriaceae	*Chloridium*	1	1	-	-	-	-	-	[16]
S	Ascomycota	Chaetothyriales	Herpotrichiellaceae	*Cladophialophora*	5	1 + sp.	-	-	-	-	-	[17]
S	Ascomycota	Chaetothyriales	Herpotrichiellaceae	*Exophiala*	7	5 + sp.	-	-	-	-	-	[16,17]
S	Ascomycota	Chaetothyriales	Incertae_Sedis	*Phaeoannellomyces*	2	1	-	-	-	-	-	[16]
S	Ascomycota	Dothideales	Dothioraceae	*Hormonema*	1	1	-	-	-	-	-	[16,17]
S	Ascomycota	Eurotiales	Aspergillaceae	*Aspergillus*	14	13 + sp.	-	-	-	-	-	[12,16]
S	Ascomycota	Eurotiales	Aspergillaceae	*Neosartorya*	1	sp.	-	-	-	-	-	[16,17]
S	Ascomycota	Eurotiales	Aspergillaceae	*Penicillago*	1	1	-	-	-	-	-	This study
S	Ascomycota	Eurotiales	Aspergillaceae	*Penicillium*	34	25 + sp.	-	-	-	-	-	[12,16,17]
S	Ascomycota	Eurotiales	Trichocomaceae	*Sagenomella*	2	2	-	-	-	-	-	[16,17]
S	Ascomycota	Eurotiales	Trichocomaceae	*Talaromyces*	6	5 + sp.	-	-	-	-	-	[12,16,17]
S	Ascomycota	Eurotiales	Trichocomaceae	*Thermomyces*	1	1	-	-	-	-	-	[16]
S	Ascomycota	Glomerellales	Plectosphaerellaceae	*Acrostalagmus*	1	1	-	-	-	-	-	[12,16,17]
S	Ascomycota	Glomerellales	Plectosphaerellaceae	*Furcasterigmium*	1	1	-	-	-	-	-	[16]
S	Ascomycota	Helotiales	Dermateaceae	*Mollisia*	1	1	-	-	-	-	-	[12]
S	Ascomycota	Helotiales	Helotiaceae	*Articulospora*	1	sp.	-	-	-	-	-	[16]
S	Ascomycota	Helotiales	Helotiaceae	*Bloxamia*	1	sp.	-	-	-	-	-	[16,17]
S	Ascomycota	Helotiales	Helotiaceae	*Crocicreas*	1	1	-	-	-	-	-	[16,17]
S	Ascomycota	Helotiales	Helotiaceae	*Scytalidium*	1	1	-	-	-	-	-	[16]
S	Ascomycota	Helotiales	Helotiaceae	*Tetracladium*	3	sp.	-	-	-	-	-	[16,17]
S	Ascomycota	Helotiales	Helotiaceae	*Tympanis*	1	sp.	-	-	-	-	-	[17]
S	Ascomycota	Helotiales	Hyaloscyphaceae	*Arachnopeziza*	1	sp.	-	-	-	-	-	[16,17]

S	Ascomycota	Helotiales	Hyaloscyphaceae	*Hyaloscypha*	2	1	-	-	-	-	-	[16,17]
S	Ascomycota	Helotiales	Incertae_Sedis	*Chalara*	1	sp.	-	-	-	-	-	[16,17]
S	Ascomycota	Hypocreales	Clavicipitaceae	*Metarhizium*	1	1	-	-	-	-	-	[12]
S	Ascomycota	Hypocreales	Hypocreaceae	*Monocillium*	2	2	-	-	-	-	-	[16,17]
S	Ascomycota	Hypocreales	Incertae_Sedis	*Sesquicillium*	1	1	-	-	-	-	-	[16,17]
S	Ascomycota	Hypocreales	Ophiocordycipitaceae	*Polycephalomyces*	1	1	-	-	-	-	-	This study
S	Ascomycota	Hypocreales	Stachybotryaceae	*Albifimbria*	1	1	-	-	-	-	-	[12]
S	Ascomycota	Hypocreales	Stachybotryaceae	*Myrothecium*	1	1	-	-	-	-	-	[12,16,17]
S	Ascomycota	Hypocreales	Stachybotryaceae	*Stachybotrys*	1	1	-	-	-	-	-	[12,17]
S	Ascomycota	Incertae_Sedis	Incertae_Sedis	*Hawksworthiana*	1	sp.	-	-	-	-	-	[16]
S	Ascomycota	Microascales	Microascaceae	*Cephalotrichum*	1	1	-	-	-	-	-	[16]
S	Ascomycota	Myrmecridiales	Myrmecridiaceae	*Myrmecridium*	1	1	-	-	-	-	-	[16,17]
S	Ascomycota	Onygenales	Arachnomycetaceae	*Arachnomyces*	1	1	-	-	-	-	-	[12,16,17]
S	Ascomycota	Onygenales	Gymnoascaceae	*Arachniotus*	1	1	-	-	-	-	-	[16,17]
S	Ascomycota	Onygenales	Gymnoascaceae	*Gymnascella*	2	1	-	-	-	-	-	[12,16,17]
S	Ascomycota	Onygenales	Gymnoascaceae	*Leucothecium*	2	1	-	-	-	-	-	[16,17]
S	Ascomycota	Onygenales	Incertae_Sedis	*Chrysosporium*	7	4	-	-	-	-	-	[12,16]
S	Ascomycota	Onygenales	Onygenaceae	*Auxarthron*	1	1	-	-	-	-	-	[16,17]
S	Ascomycota	Onygenales	Onygenaceae	*Myriodontium*	1	1	-	-	-	-	-	[16,17]
S	Ascomycota	Pezizales	Ascobolaceae	*Ascobolus*	1	sp.	-	-	-	-	-	[16,17]
S	Ascomycota	Pezizales	Discinaceae	*Gyromitra*	1	sp.	-	-	-	-	-	[16,17]
S	Ascomycota	Pleosporales	Amorosiaceae	*Angustimassarina*	1	1	-	-	-	-	-	[12,16]
S	Ascomycota	Pleosporales	Biatriosporaceae	*Biatriospora*	1	1	-	-	-	-	-	This study
S	Ascomycota	Pleosporales	Coniothyriaceae	*Coniothyrium*	1	1	-	-	-	-	-	[17]
S	Ascomycota	Pleosporales	Cucurbitariaceae	*Neocucurbitaria*	2	2	-	-	-	-	-	[16]
S	Ascomycota	Pleosporales	Cucurbitariaceae	*Pyrenochaeta*	1	sp.	-	-	-	-	-	[16,17]

S	Ascomycota	Pleosporales	Cucurbitariaceae	*Pyrenochaetopsis*	6	2 + sp.	-	-	-	-	-	[17]
S	Ascomycota	Pleosporales	Didymellaceae	*Phoma*	2	sp.	-	-	-	-	-	[12,17]
S	Ascomycota	Pleosporales	Didymosphaeriaceae	*Didymosphaeria*	1	1	-	-	-	-	-	[12,16,17]
S	Ascomycota	Pleosporales	Didymosphaeriaceae	*Paraphaeosphaeria*	3	2 + sp.	-	-	-	-	-	[16,17]
S	Ascomycota	Pleosporales	Didymosphaeriaceae	*Pseudocamarosporium*	1	1	-	-	-	-	-	[12,16,17]
S	Ascomycota	Pleosporales	Lentitheciaceae	*Keissleriella*	1	1	-	-	-	-	-	[16,17]
S	Ascomycota	Pleosporales	Lentitheciaceae	*Poaceascoma*	1	1	-	-	-	-	-	[16]
S	Ascomycota	Pleosporales	Lophiostomataceae	*Lophiostoma*	1	1	-	-	-	-	-	[12,16,17]
S	Ascomycota	Pleosporales	Lophiotremataceae	*Lophiotrema*	2	1 + sp.	-	-	-	-	-	[12,16,17]
S	Ascomycota	Pleosporales	Massarinaceae	*Massarina*	1	1	-	-	-	-	-	[12,17]
S	Ascomycota	Pleosporales	Phaeosphaeriaceae	*Hydeomyces*	4	1	-	-	-	-	-	[16,17]
S	Ascomycota	Pleosporales	Phaeosphaeriaceae	*Neosetophoma*	4	2 + sp.	-	-	-	-	-	[16,17]
S	Ascomycota	Pleosporales	Pyrenochaetopsidaceae	*Neopyrenochaeta*	1	1	-	-	-	-	-	[16]
S	Ascomycota	Pleosporales	Sporormiaceae	*Preussia*	1	1	-	-	-	-	-	[12,16,17]
S	Ascomycota	Pleosporales	Torulaceae	*Torula*	1	1	-	-	-	-	-	[16]
S	Ascomycota	Saccharomycetales	Debaryomycetaceae	*Debaryomyces*	1	1	-	-	-	-	-	[16,17]
S	Ascomycota	Saccharomycetales	Incertae_Sedis	*Candida*	2	2	-	-	-	-	-	[16,17]
S	Ascomycota	Saccharomycetales	Incertae_Sedis	*Starmerella*	1	1	-	-	-	-	-	[16]
S	Ascomycota	Saccharomycetales	Lipomycetaceae	*Lipomyces*	2	2	-	-	-	-	-	[16]
S	Ascomycota	Saccharomycetales	Pichiaceae	*Kregervanrija*	1	1	-	-	-	-	-	[16]
S	Ascomycota	Saccharomycetales	Trichomonascaceae	*Trichomonascus*	1	sp.	-	-	-	-	-	[16]
S	Ascomycota	Saccharomycetales	Unknown	*Unknown*	1	1	-	-	-	-	-	This study
S	Ascomycota	Sordariales	Chaetomiaceae	*Chaetomium*	1	sp.	-	-	-	-	-	[12,16]
S	Ascomycota	Sordariales	Chaetomiaceae	*Humicola*	3	2 + sp.	-	-	-	-	-	[12,16]
S	Ascomycota	Sordariales	Chaetomiaceae	*Myceliophthora*	1	1	-	-	-	-	-	This study
S	Ascomycota	Thelebolales	Pseudeurotiaceae	*Geomyces*	2	1 + sp.	-	-	-	-	-	[12,16,17]

S	Ascomycota	Thelebolales	Pseudeurotiaceae	*Gymnostellatospora*	2	1	-	-	-	-	-	[16,17]
S	Ascomycota	Thelebolales	Pseudeurotiaceae	*Pseudeurotium*	6	3 + sp.	-	-	-	-	-	[16,17]
S	Ascomycota	Thelebolales	Pseudeurotiaceae	*Pseudogymnoascus*	5	2 + sp.	-	-	-	-	-	[12,16,17]
S	Ascomycota	Tubeufiales	Tubeufiaceae	*Titaea*	1	1	-	-	-	-	-	[16,17]
S	Ascomycota	Unknown	Unknown	Unknown	1	1	-	-	-	-	-	This study
S	Ascomycota	Xylariales	Xylariaceae	*Zygosporium*	1	1	-	-	-	-	-	[16]
S	Basidiomycota	Agaricales	Marasmiaceae	*Marasmius*	1	1	-	-	-	-	-	[12,16,17]
S	Basidiomycota	Agaricales	Omphalotaceae	*Rhodocollybia*	1	1	-	-	-	-	-	[16,17]
S	Basidiomycota	Agaricales	Psathyrellaceae	*Coprinellus*	2	1	-	-	-	-	-	[12,16,17]
S	Basidiomycota	Agaricales	Tricholomataceae	*Clitocybe*	1	1	-	-	-	-	-	[16]
S	Basidiomycota	Cystofilobasidiales	Cystofilobasidiaceae	*Cystofilobasidium*	4	2	-	-	-	-	-	[16,17]
S	Basidiomycota	Cystofilobasidiales	Mrakiaceae	*Tausonia*	1	1	-	-	-	-	-	[16]
S	Basidiomycota	Filobasidiales	Filobasidiaceae	*Filobasidium*	4	4	-	-	-	-	-	[16,17]
S	Basidiomycota	Filobasidiales	Filobasidiaceae	*Naganishia*	7	3	-	-	-	-	-	[16]
S	Basidiomycota	Filobasidiales	Piskurozymaceae	*Solicoccozyma*	6	3	-	-	-	-	-	[16]
S	Basidiomycota	Geminibasidiales	Geminibasidiaceae	*Geminibasidium*	1	1	-	-	-	-	-	[16,17]
S	Basidiomycota	Holtermanniales	Incertae_Sedis	*Holtermanniella*	1	1	-	-	-	-	-	[16]
S	Basidiomycota	Incertae_Sedis	Buckleyzymaceae	*Buckleyzyma*	1	sp.	-	-	-	-	-	[16,17]
S	Basidiomycota	Incertae_Sedis	Incertae_Sedis	*Curvibasidium*	1	1	-	-	-	-	-	[16]
S	Basidiomycota	Sporidiobolales	Sporidiobolaceae	*Rhodotorula*	1	1	-	-	-	-	-	[12,16,17]
S	Basidiomycota	Tremellales	Bulleraceae	*Pseudotremella*	1	1	-	-	-	-	-	[16]
S	Basidiomycota	Tremellales	Bulleribasidiaceae	*Dioszegia*	1	1	-	-	-	-	-	[16]
S	Basidiomycota	Tremellales	Bulleribasidiaceae	*Hannaella*	2	2	-	-	-	-	-	[16]
S	Basidiomycota	Tremellales	Bulleribasidiaceae	*Vishniacozyma*	6	3 + sp.	-	-	-	-	-	[16]
S	Basidiomycota	Tremellales	Cryptococcaceae	*Kwoniella*	2	1 + sp.	-	-	-	-	-	[16]
S	Basidiomycota	Tremellales	Tremellaceae	*Cryptococcus*	1	sp.	-	-	-	-	-	[12,16,17]

S	Basidiomycota	Tremellales	Trimorphomycetaceae	*Saitozyma*	2	1	-	-	-	-	-	[16]
S	Basidiomycota	Trichosporonales	Trichosporonaceae	*Apiotrichum*	1	1	-	-	-	-	-	[16,17]
S	Calcarisporiellomycota	Calcarisporiellales	Calcarisporiellaceae	*Calcarisporiella*	1	sp.	-	-	-	-	-	[16]
S	Mortierellomycota	Mortierellales	Mortierellaceae	*Mortierella*	45	15 + sp.	-	-	-	-	-	[12,16,17]
S	Mucoromycota	Mucorales	Mucoraceae	*Actinomucor*	1	1	-	-	-	-	-	[12,16,17]
S	Mucoromycota	Mucorales	Mucoraceae	*Mucor*	3	3	-	-	-	-	-	[12,16]
S	Mucoromycota	Mucorales	Unknown	Unknown	1	1	-	-	-	-	-	This study
S	Mucoromycota	Umbelopsidales	Umbelopsidaceae	*Umbelopsis*	7	1 + sp.	-	-	-	-	-	[12,16,17]
**Total saprotrophs**					**300 ASVs**							
P	Ascomycota	Botryosphaeriales	Botryosphaeriaceae	*Diplodia*	1	sp.	-	+	-	-	-	[3,12]
P	Ascomycota	Botryosphaeriales	Botryosphaeriaceae	*Dothiorella*	1	1	-	+	-	-	-	[3,12]
P	Ascomycota	Botryosphaeriales	Botryosphaeriaceae	*Neofusicoccum*	1	1	-	+	-	-	-	[3,12]
P	Ascomycota	Capnodiales	Mycosphaerellaceae	*Septoria*	2	1	+	-	-	-	-	[12,16]
P	Ascomycota	Capnodiales	Teratosphaeriaceae	*Devriesia*	2	1 + sp.	+	-	-	-	-	[16,17]
P	Ascomycota	Chaetothyriales	Incertae_Sedis	*Neophaeococcomyces*	1	1	+	-	-	-	-	[16]
P	Ascomycota	Chaetothyriales	Trichomeriaceae	*Knufia*	3	1	+	-	-	-	-	[16]
P	Ascomycota	Diaporthales	Valsaceae	*Phomopsis*	1	1	-	+	-	-	-	[3]
P	Ascomycota	Glomerellales	Plectosphaerellaceae	*Chordomyces*	2	1	+	-	-	-	-	[17]
P	Ascomycota	Glomerellales	Plectosphaerellaceae	*Gibellulopsis*	2	1 + sp.	+	-	-	-	-	[17]
P	Ascomycota	Glomerellales	Plectosphaerellaceae	*Plectosphaerella*	2	2	+	-	-	-	-	[16,17]
P	Ascomycota	Helotiales	Dermateaceae	*Neofabraea*	1	1	+	-	-	-	-	[16,17]
P	Ascomycota	Helotiales	Dermateaceae	*Trichosporiella*	1	1	+	-	-	-	-	[17]
P	Ascomycota	Helotiales	Helotiaceae	*Pseudopezicula*	1	1	+	-	-	-	-	[16,17]
P	Ascomycota	Helotiales	Incertae_Sedis	*Cadophora*	2	2	-	+	-	-	-	[3,12]
P	Ascomycota	Helotiales	Sclerotiniaceae	*Botrytis*	1	1	+	-	-	-	-	[16,17]
P	Ascomycota	Helotiales	Sclerotiniaceae	*Stromatinia*	1	1	+	-	-	-	-	[16,17]
P	Ascomycota	Hypocreales	Bionectriaceae	*Clonostachys*	1	1	+	-	-	-	-	[12,17]

P	Ascomycota	Hypocreales	Incertae_Sedis	*Acremonium*	4	3 + sp.	+	-	-	-	-	[12]
P	Ascomycota	Hypocreales	Incertae_Sedis	*Fusariella*	1	sp.	+	-	-	-	-	[16]
P	Ascomycota	Hypocreales	Incertae_Sedis	*Sarocladium*	2	2	+	-	-	-	-	[16]
P	Ascomycota	Hypocreales	Nectriaceae	*Fusarium*	8	7	+	-	-	-	-	[12,16]
P	Ascomycota	Hypocreales	Nectriaceae	Unknown	1	1	+	-	-	-	-	This study
P	Ascomycota	Mytilinidales	Kirschsteiniotheliaceae	*Taeniolella*	1	1	+	-	-	-	-	[17]
P	Ascomycota	Phaeomoniellales	Phaeomoniellaceae	*Phaeomoniella*	29	1	-	+	-	-	-	[3,12]
P	Ascomycota	Pleosporales	Didymellaceae	*Boeremia*	1	1	+	-	-	-	-	[12,16,17]
P	Ascomycota	Pleosporales	Didymellaceae	*Chaetasbolisia*	1	1	+	-	-	-	-	[17]
P	Ascomycota	Pleosporales	Didymellaceae	*Didymella*	11	3	+	-	-	-	-	[16,17]
P	Ascomycota	Pleosporales	Didymellaceae	*Neodidymelliopsis*	1	1	+	-	-	-	-	[17]
P	Ascomycota	Pleosporales	Didymosphaeriaceae	*Kalmusia*	2	1 + sp.	-	+	-	-	-	[31]
P	Ascomycota	Pleosporales	Incertae_Sedis	*Foliophoma*	1	1	+	-	-	-	-	[16]
P	Ascomycota	Pleosporales	Leptosphaeriaceae	*Leptosphaeria*	1	1	+	-	-	-	-	[16,17]
P	Ascomycota	Pleosporales	Phaeosphaeriaceae	*Chaetosphaeronema*	1	sp.	+	-	-	-	-	[16,17]
P	Ascomycota	Pleosporales	Phaeosphaeriaceae	*Paraphoma*	5	3	+	-	-	-	-	[16,17]
P	Ascomycota	Pleosporales	Phaeosphaeriaceae	*Populocrescentia*	1	1	+	-	-	-	-	[17]
P	Ascomycota	Pleosporales	Phaeosphaeriaceae	*Sclerostagonospora*	1	sp.	-	+	-	-	-	[12,16]
P	Ascomycota	Pleosporales	Phaeosphaeriaceae	*Setophaeosphaeria*	1	1	+	-	-	-	-	[16,17]
P	Ascomycota	Pleosporales	Phaeosphaeriaceae	Unknown	2	2	+	-	-	-	-	This study
P	Ascomycota	Pleosporales	Pleosporaceae	*Alternaria*	7	4 + sp.	+	-	-	-	-	[12,16,17]
P	Ascomycota	Pleosporales	Pleosporaceae	*Curvularia*	5	4 + sp.	+	-	-	-	-	[16,17]
P	Ascomycota	Pleosporales	Pleosporaceae	*Stemphylium*	1	1	+	-	-	-	-	[16,17]
P	Ascomycota	Pleosporales	Unknown	Unknown	1	1	+	-	-	-	-	This study
P	Ascomycota	Togniniales	Togniniaceae	*Phaeoacremonium*	8	2	-	+	-	-	-	[3,12,16,17]
P	Ascomycota	Venturiales	Venturiaceae	*Tyrannosorus*	1	1	+	-	-	-	-	[17]
P	Ascomycota	Venturiales	Venturiaceae	*Venturia*	1	sp.	+	-	-	-	-	[16,17]
P	Ascomycota	Xylariales	Amphisphaeriaceae	*Seimatosporium*	2	1	-	+	-	-	-	[12,16]

P	Ascomycota	Xylariales	Bartaliniaceae	*Truncatella*	1	1	-	+	-	-	-	[12,16,17]
P	Ascomycota	Xylariales	Diatrypaceae	*Cryptovalsa*	1	1	-	+	-	-	-	[3,12,16,17]
P	Ascomycota	Xylariales	Diatrypaceae	*Eutypa*	1	1	-	+	-	-	-	[3,12,16,17]
P	Ascomycota	Xylariales	Microdochiaceae	*Microdochium*	4	2 + sp.	+	-	-	-	-	[16]
P	Basidiomycota	Agaricales	Incertae_Sedis	*Uncobasidium*	1	sp.	+	-	+	-	-	[16]
P	Basidiomycota	Agaricales	Omphalotaceae	*Gymnopus*	4	2	+	-	+	-	-	[17]
P	Basidiomycota	Agaricales	Tricholomataceae	*Mycena*	4	1 + sp.	+	-	-	-	-	[17]
P	Basidiomycota	Cystofilobasidiales	Mrakiaceae	*Itersonilia*	1	1	+	-	-	-	-	[16,17]
P	Basidiomycota	Hymenochaetales	Hymenochaetaceae	*Fomitiporia*	16	1 + sp.	-	+	+	-	-	[3,12,16,17]
P	Basidiomycota	Hymenochaetales	Hymenochaetaceae	*Phellinus*	8	2	-	+	+	-	-	[12,16,17]
P	Basidiomycota	Hymenochaetales	Hymenochaetaceae	Unknown	53	-	-	+	+	-	-	[8]
P	Basidiomycota	Incertae_Sedis	Incertae_Sedis	*Xenasmatella*	1	1	+	-	+	-	-	[16,17]
P	Basidiomycota	Polyporales	Ganodermataceae	*Ganoderma*	1	1	+	-	+	-	-	[16,17]
P	Basidiomycota	Russulales	Peniophoraceae	*Peniophora*	2	2	+	-	+	-	-	[16,17]
P	Olpidiomycota	Olpidiales	Olpidiaceae	*Olpidium*	1	2	+	-	-	-	-	[17]
**Total pathotrophs**					**226 ASVs**							
	**Total plant-pathogens**						**98 ASVs**					
	**Total GTD-pathotrophs**							**128 ASVs**				
	**Total causing white rot**								**86 ASVs**			
Sy	Ascomycota	Helotiales	Incertae_Sedis	*Leohumicola*	2	1 + sp.	-	-	-	-	+	[16,17]
Sy	Ascomycota	Helotiales	Myxotrichaceae	*Oidiodendron*	8	5	-	-	-	-	+	[17]
Sy	Ascomycota	Mytilinidales	Gloniaceae	*Cenococcum*	3	1 + sp.	-	-	-	+	-	[16,17]
Sy	Ascomycota	Pezizales	Pezizaceae	*Terfezia*	1	1	-	-	-	+	-	[16,17]
Sy	Basidiomycota	Agaricales	Cortinariaceae	*Cortinarius*	7	4 + sp.	-	-	-	+	-	[16,17]
Sy	Basidiomycota	Agaricales	Hygrophoraceae	*Hygrophorus*	1	1	-	-	-	+	-	[16,17]
Sy	Basidiomycota	Agaricales	Hymenogastraceae	*Hymenogaster*	1	1	-	-	-	+	-	[16,17]
Sy	Basidiomycota	Agaricales	Inocybaceae	*Inocybe*	2	1 + sp.	-	-	-	+	-	[16,17]
Sy	Basidiomycota	Agaricales	Tricholomataceae	*Mycenella*	1	1	-	-	-	+	-	[17]

Sy	Basidiomycota	Agaricales	Tricholomataceae	*Tricholoma*	1	1	-	-	-	+	-	[16,17]
Sy	Basidiomycota	Atheliales	Atheliaceae	*Amphinema*	2	1	-	-	-	+	-	[16,17]
Sy	Basidiomycota	Atheliales	Atheliaceae	*Piloderma*	1	sp.	-	-	-	+	-	[16,17]
Sy	Basidiomycota	Boletales	Boletaceae	*Leccinellum*	2	1	-	-	-	+	-	[16,17]
Sy	Basidiomycota	Boletales	Melanogastraceae	*Melanogaster*	1	1	-	-	-	+	-	[16]
Sy	Basidiomycota	Cantharellales	Clavulinaceae	*Clavulina*	8	sp.	-	-	-	+	-	[16,17]
Sy	Basidiomycota	Cantharellales	Hydnaceae	*Hydnum*	2	1 + sp.	-	-	-	+	-	[16,17]
Sy	Basidiomycota	Russulales	Russulaceae	*Lactarius*	2	2	-	-	-	+	-	[16,17]
Sy	Basidiomycota	Russulales	Russulaceae	*Russula*	12	8 + sp.	-	-	-	+	-	[17]
Sy	Basidiomycota	Sebacinales	Sebacinaceae	*Sebacina*	2	sp.	-	-	-	+	-	[16,17]
Sy	Basidiomycota	Thelephorales	Bankeraceae	*Hydnellum*	1	1	-	-	-	+	-	[16,17]
Sy	Basidiomycota	Thelephorales	Thelephoraceae	*Pseudotomentella*	1	1	-	-	-	+	-	[16,17]
Sy	Basidiomycota	Thelephorales	Thelephoraceae	*Thelephora*	3	2 + sp.	-	-	-	+	-	[16,17]
Sy	Basidiomycota	Thelephorales	Thelephoraceae	*Tomentella*	2	2	-	-	-	+	-	[16,17]
**Total symbiotrophs**					**66 ASVs**							
	Ascomycota	Dothideales	Aureobasidiaceae	*Aureobasidium*	21	1 + sp.	-	-	-	-	-	[2,9,30]
BCA	Ascomycota	Hypocreales	Clavicipitaceae	*Keithomyces*	1	1	-	-	-	-	-	[17]
BCA	Ascomycota	Hypocreales	Clavicipitaceae	*Metapochonia*	1	1	-	-	-	-	-	[17]
BCA	Ascomycota	Hypocreales	Hypocreaceae	*Trichoderma*	3	3	-	-	-	-	-	[9,16]
BCA	Ascomycota	Hypocreales	Nectriaceae	*Fusicolla*	1	1	-	-	-	-	-	[16]
BCA	Basidiomycota	Tremellales	Rhynchogastremataceae	*Papiliotrema*	2	2	-	-	-	-	-	[16]
BCA	Basidiomycota	Tremellales	Tremellaceae	*Tremella*	1	sp.	-	-	-	-	-	[16]
**Total potencial BCAs**					**30 ASVs**							
